# Exploration of the Nurse Shark (*Ginglymostoma cirratum*) Plasma Immunoproteome Using High-Resolution LC-MS/MS

**DOI:** 10.3389/fimmu.2022.873390

**Published:** 2022-06-06

**Authors:** Fiona K. Bakke, Manu Kumar Gundappa, Hanover Matz, David A. Stead, Daniel J. Macqueen, Helen Dooley

**Affiliations:** ^1^School of Biological Sciences, University of Aberdeen, Aberdeen, United Kingdom; ^2^The Roslin Institute and Royal (Dick) School of Veterinary Studies, University of Edinburgh, Edinburgh, United Kingdom; ^3^Department of Microbiology and Immunology, Institute of Marine and Environmental Technology (IMET), University of Maryland School of Medicine, Baltimore, MD, United States; ^4^Aberdeen Proteomics, The Rowett Institute, University of Aberdeen, Aberdeen, United Kingdom

**Keywords:** cartilaginous fishes (Chondrichthyes), shark, plasma, proteome, immunoglobulin, *de novo* transcriptome

## Abstract

Many animals of scientific importance lack species-specific reagents (e.g., monoclonal antibodies) for in-depth studies of immune proteins. Mass spectrometry (MS)-based proteomics has emerged as a useful method for monitoring changes in protein abundance and modifications in non-model species. It can be used to quantify hundreds of candidate immune molecules simultaneously without the generation of new reagents. Here, we used MS-based proteomics to identify and quantify candidate immune proteins in the plasma of the nurse shark (*Ginglymostoma cirratum*), a cartilaginous fish and representative of the most basal extant vertebrate lineage with an immunoglobulin-based immune system. Mass spectrometry-based LC-MS/MS was performed on the blood plasma of nurse sharks immunized with human serum albumin (n=4) or sham immunized (n=1), and sampled at days 0 (baseline control), 1, 2, 3, 5, 7, 14, 21, 28, 25, 42 and 49. An antigen-specific antibody response was experimentally confirmed post-immunization. To provide a high-quality reference to identify proteins, we assembled and annotated a multi-tissue *de novo* transcriptome integrating long- and short-read sequence data. This comprised 62,682 contigs containing open reading frames (ORFs) with a length >80 amino acids. Using this transcriptome, we reliably identified 626 plasma proteins which were broadly categorized into coagulation, immune, and metabolic functional groups. To assess the feasibility of performing LC-MS/MS proteomics in nurse shark in the absence of species-specific protein annotations, we compared the results to an alternative strategy, mapping peptides to proteins predicted in the genome assembly of a related species, the whale shark (*Rhincodon typus*). This approach reliably identified 297 proteins, indicating that useful data on the plasma proteome may be obtained in many instances despite the absence of a species-specific reference protein database. Among the plasma proteins defined against the nurse shark transcriptome, fifteen showed consistent changes in abundance across the immunized shark individuals, indicating a role in the immune response. These included alpha-2-macroglobulin (A2M) and a novel protein yet to be characterized in diverse vertebrate lineages. Overall, this study enhances genetic and protein-level resources for nurse shark research and vastly improves our understanding of the elasmobranch plasma proteome, including its remodelling following immune stimulation.

## Background

Few species-specific research tools are available for the study of immune protein responses in many scientifically important taxa. Included here are the cartilaginous fishes, the oldest extant vertebrate lineage to possess an adaptive immune system based on immunoglobulins (Igs) (reviewed by 1). Only a small number of cartilaginous fish-specific monoclonal antibodies (mAbs) have been generated to date, mainly targeting Ig heavy and light chains (e.g., 2–4). Further, due to the large evolutionary distances involved, mAbs raised against immune proteins from mammals rarely cross-react with cartilaginous fish proteins. This issue is further compounded by the marked differences in immune gene family repertoires often observed when comparing cartilaginous fish with other taxa (e.g., 5, 6). Considering the high cost of developing and validating custom mAbs, this strategy does not offer an efficient solution for investigations of immune proteins in cartilaginous fishes and many other taxa.

High-resolution proteomics on liquid chromatography-tandem mass spectrometry (LC-MS/MS) platforms is increasingly used to study quantitative changes in protein abundance. For instance, LC-MS/MS has been used to characterize human plasma proteomes (7, 8), and to assist in the identification of biomarkers for diseases such as cancer (e.g., 9, 10). Such tools have also been applied to characterize proteomes in non-mammalian species (e.g., 11–14), permitting the identification and quantification of many proteins simultaneously and circumventing the need for specific mAbs (reviewed by 15). However, such methods require a comprehensive sequence database to match the enzymatically digested peptides detected during LC-MS/MS back to their original proteins (16–19).

Vertebrate blood plasma provides a medium for the transport of proteins fundamental to many key functions including immunity, metabolism, and blood clotting. Many of these circulating proteins derive from other tissues, with their levels in plasma informing on processes occurring elsewhere (20, 21). Consequently, plasma proteomics offers a useful approach to inform on immunological functions. To date, very little is known about the plasma proteins of cartilaginous fishes or their individual contribution to immune defence, with studies primarily addressing their identification and evolution at the genomic level rather than their presence in plasma and associated immune responsiveness (e.g., 22–24). Where functional studies have been performed, these have focused on Igs (e.g., 4, 25) or individual proteins that are present at high abundance in shark plasma, e.g., haptoglobin and hemopexin (6, 26). To address this knowledge gap, we performed high resolution LC-MS/MS proteomics on 60 longitudinally collected plasma samples from five immunized nurse sharks along with a sham immunized control. We also generated a high-quality *in silico* proteome for this species *via* the assembly of a novel transcriptome built with PacBio and Illumina data. Our previous immunization study on rainbow trout (*Oncorhynchus mykiss*), also using LC-MS/MS proteomics (14), identified abundance changes in 278 plasma proteins, including both classical immune proteins (e.g., complement components) and proteins not usually associated with immune responses in mammals (e.g., apolipoproteins). A separate study by Morro and colleagues (27) identified a greater number of plasma proteins (1822) in rainbow trout using LC-MS/MS label-free proteomics in combination with enrichment of low abundance proteins. We therefore hypothesized that the approach used in our previous study would yield similar results in terms of the number and types of immune proteins detected in nurse shark. Indeed, our approach led to the reliable detection, identification, and measurement of concurrent abundance changes in 260 nurse shark plasma proteins, thereby extending our knowledge of the molecules comprising shark plasma and their responses following immunization.

## Materials and Methods

### RNA Extraction and Transcriptome Sequencing

All animal work was performed in accordance with the University of Maryland, School of Medicine Institutional Animal Care and Use Committee (IACUC) approved protocol. One wild-caught juvenile (female, aged 1-3 years) nurse shark was euthanized by overdose in tricaine methanesulfonate (MS-222). Samples of 7 tissues (spleen, liver, epigonal, brain, gill, spiral valve, and kidney) were taken and stored in RNALater (Life Technologies, USA). Total RNA was extracted from each sample using the Qiagen RNeasy Mini Kit according to the manufacturer’s instructions. Briefly, 2 samples of each tissue were lysed and homogenized in 1 ml QIAzol Lysis Reagent using the Qiagen TissueLyser II. Phase separation was achieved through the addition of 0.2 ml chloroform to each tube, followed by centrifugation for 10 min at 13,000 rpm and 4°C. The aqueous phase was removed, and an equivalent volume of 70% ethanol added. Total RNA was isolated by adding this solution to Qiagen RNeasy Mini spin columns, washing once with 700 μl RW1 buffer and once with 500 μl RPE buffer, centrifuging at 13,000 rpm for 15 sec between steps. A final wash was carried out by adding 500 μl RPE buffer, centrifuging at 13,000 rpm for 2 min prior to transfer of the column to a clean tube and elution of the RNA in 40μl RNase-free water. RNA quality and concentration was assessed using a Qubit 3.0 fluorometer and RNA Broad-Range Assay kit (Thermo Fisher Scientific, Waltham, MA, USA). RNA integrity was assessed using an Agilent Bioanalyzer. Sequencing-library preparation and sequencing were performed by the Institute for Genome Sciences, University of Maryland, Baltimore, USA, using the Illumina HiSeq 4000 (PE150) and PacBio Sequel platforms. RNA from each tissue was indexed and sequenced using two Illumina lanes per tissue, while all tissues were pooled at equimolar concentrations before sequencing using 4 SMRT cells.

### *De-Novo* Transcriptome Assembly and Annotation

Raw Illumina reads were subjected to initial quality control analysis using FastQC (version 0.11.3) (28) and then further trimmed using Trim Galore (version 0.4.0) (https://github.com/FelixKrueger/TrimGalore). A minimum length cut-off of 20 base pairs (bp) was used to trim the 3’ ends before adapter removal, and a Phred score cut-off of 25 was applied. The remaining sequences were combined into one set of paired-end reads and normalized using Trinity *in silico* normalization (29).

Raw PacBio reads were assembled, clustered and polished using the IsoSeq3 low-level workflow pipeline (https://github.com/PacificBiosciences/IsoSeq/blob/master/READMEv3.2.md). Consensus sequences were generated from subread alignments for all zero mode waveguides (ZMW) with at least one full pass. The *Lima* module was then used to remove primers and barcodes and demultiplex the reads, providing a sequence dataset. Sequences were refined using the *Refine* module, where poly(A) tails and concatemers were removed. The resulting sequences were merged into a single set prior to clustering, using the *Cluster* algorithm, to provide a non-redundant set of transcripts. The *Polish* option was then used to resolve any remaining gaps in the transcript set.

*De novo* transcriptome assembly was carried out using Trinity (version 2.0.6) (29) using the *–long_reads <string>* option to combine the polished IsoSeq 3 read output with the trimmed Illumina reads. The minimum contig length was set to 100 since the maximum Illumina sequence length post-trimming was 151 bp. The resultant hybrid assembly was filtered for contigs with a minimum expression of 1 transcript per million (TPM) using a standard protocol on Trinity (https://github.com/trinityrnaseq/trinityrnaseq/wiki/Trinity-Transcript-Quantification#filtering-transcripts). The TPM filtered hybrid assembly and polished IsoSeq3 assembly were merged to retain any isoforms absent from the hybrid assembly. The pooled transcripts were clustered and collapsed using *cd-hit* (30, 31) with a minimum identity cut-off of 99%. Transdecoder (https://github.com/TransDecoder/TransDecoder/wiki) was used to predict open reading frames (ORFs) encoding a minimum of 80 amino acids. Only the longest ORF was retained for each transcript. All the protein sequences were subjected to BLASTp to identify protein matches against the UniProt protein database, with the Pfam v.32.0 database (32) used to detect protein domains. The outputs from these two steps were used to predict transcripts with coding potential using *Transdecoder.predict*. Annotation of the final set of transcripts was performed using EnTAP (33). The Benchmarking Universal Single-Copy Orthologues (BUSCO) v.3.0.2 (34, 35) tool was used to indicate the degree of completeness of the transcriptome against the vertebrata Odb10 database.

### Nurse Shark Immunizations and Plasma Sampling

Five wild-caught nurse sharks, aged between 2-3 years and weighing between 1.4-1.7 kg, were obtained from Florida coastal waters under a Special Activity License from the Florida Fish and Wildlife Conservation Commission. The animals were flown to Baltimore, where they were maintained in a 12,000L tank containing continuously recirculating sea water at 28°C at the Institute of Marine and Environmental Technology (IMET), Baltimore, USA. Animals were acclimatized for at least 3 months prior to sampling/immunization and all experimental procedures were conducted in accordance with University of Maryland, School of Medicine Institutional Animal Care and Use Committee (IACUC) approved protocols.

Four sharks (three females, one male) were immunized subcutaneously into the ventral face of the lateral fin with 250 μg HSA, emulsified in an equal volume of Complete Freund’s Adjuvant (CFA). Since legal restrictions precluded the use of more than 5 sharks, we opted to sham-immunize a single shark (female) with phosphate-buffered saline (PBS) to serve as a control. Blood samples (0.2 ml) were taken from the caudal vein immediately prior to immunization on day 0, then again on days 1, 2, 3, 5, 7, 14, 21, 28, 35, 42, and 49; animals were anaesthetized in MS-222 prior to each procedure as per the approved IACUC protocol. The chosen sampling points aimed to capture (a) the earliest changes in abundance of proteins associated with the innate immune response (days 1-7), and (b) to assess changes in abundance of other proteins as the immune response progressed towards the adaptive phase (days 14-49). Shark adaptive immune responses are much slower than mammals, generally requiring at least one booster immunization and taking 3-4 months to peak (4). Thus, after the final sampling point for this study on day 49, the HSA-immunized animals received 4 additional boosts then were re-sampled on day 233. Blood samples were added to 20 μl sterile sodium citrate to prevent clotting and spun at 1000 rpm for 10 min to separate the blood constituents. Plasma was aliquoted into low protein-binding 1.5ml tubes (Thermo Fisher Scientific, Waltham, MA, USA), flash frozen, and stored at -80°C.

### Measurement of Antigen-Specific IgM

To confirm that the HSA/CFA and sham immunizations had been successful, antigen-binding ELISAs were performed using blood plasma sampled on day 0, day 49 (the final sampling point for this study) and day 233 to measure HSA-specific IgM titers for each animal. Briefly, 96 well microtiter plates were coated with 10 μg/ml HSA or 5% (w/v) milk in PBS at 100 μl per well for 1 h at room temperature and then blocked with 5% milk solution. Plasma was diluted 1:30 in PBS and a 1:3 serial dilution series set up on each plate. Samples of 100 μl/well were incubated for 2-3 h at room temperature. Anti-nurse shark IgM mouse monoclonal LK14 supernatant was diluted in PBS and 100 μl/well added to wells. Sheep anti-mouse IgG (whole molecule) peroxidase conjugate, diluted 1:1000 in PBS was also added at 100 μl/well. Plates were developed with 100 μl/well tetramethyl benzidine (TMB) substrate. After 5 mins the reaction was stopped by the addition of an equal volume of 1M H_2_SO_4_ and read at 450 nm on a SpectraMax M5 plate reader (Molecular Devices Corp, USA).

### LC-MS/MS

Plasma samples from the five, repeatedly sampled nurse sharks were prepared for proteomic analysis at the University of Aberdeen Proteomics facility, as detailed in 14. Briefly, 1 μl of plasma was diluted with 99 µl 50 mM ammonium bicarbonate and proteins were reduced in 2 mM dithiothreitol for 25 min at 60°C, S-alkylated in 4 mM iodoacetamide for 30 min at 25°C in the dark, digested with porcine trypsin (Promega) overnight at 37°C, then freeze-dried. The protein pellets were dissolved in 40 µl 0.1% TFA and desalted using ZipTip µ-C18 stage tips (Merck Millipore) following the manufacturer’s instructions. The eluted peptide solutions were dried and dissolved in 10 µl LC-MS/MS loading solvent (98 parts UHQ water: 2 parts acetonitrile: 0.1 parts formic acid). Samples were transferred to a 96-well microtitre plate ready for injection into an UltiMate 3000 RSLCnano LC system (Thermo Scientific Dionex) coupled to a Q Exactive Hybrid Quadrupole Orbitrap MS system (Thermo Scientific). The LC was configured for pre-concentration onto a PepMap RSLC C18 50 µm x 25 cm column (Thermo Scientific P/N ES802) fitted to an EASY-Spray ion source (Thermo Scientific). The loading pump solvent was UHQ water: acetonitrile: formic acid (98: 2: 0.1) at a flow rate of 10 µl/min; nano pump solvent A was UHQ water: formic acid (100: 0.1); nano pump solvent B was acetonitrile: UHQ water: formic acid (80: 20: 0.1). The LC gradient was programmed to increase the proportion of solvent B from 3-10% between 5-15 min, from 10-40% between 15-95 min, from 40-80% from 95-100 min and hold for 10 min before re-equilibration of the nano-column in 3% solvent B for 25 min. MS data acquisition was started at 10 min into the LC gradient, 5 min after switching the flow through the pre-column and continued for a total of 100 min.

### Computational Proteomics

MS data were uploaded to MaxQuant v1.5.3.30 (36). As remains the case for many species, comprehensive protein annotations (e.g., derived from a reference genome or transcriptome) were not available for the nurse shark, necessitating the generation of our *de novo* transcriptome. However, the generation of high quality genomic and/or transcriptomic assemblies is both costly and non-trivial. We were therefore interested to establish what quality of data would be returned using a cross-species reference for protein identification, assuming the hypothetical absence of a species-specific reference. We therefore compared the number of quantitative protein identifications obtained against a database predicted from the genome of a related species, the whale shark (NCBI accession ASM164234v2; 37), to those obtained against our new species-specific transcriptome. At the time of this study, the whale shark represented the closest relative to the nurse shark for which an annotated genome was available. However, although nurse and whale sharks are members of the same Order (Orectolobiformes), they occupy different Families (*Ginglymostomatidae* and *Rhincodontidae*, respectively).

The Andromeda peptide search engine within MaxQuant (17) was used to match the MS of all detected peptides against the two reference protein databases. Digestion type was “trypsin”, two missed cleavages were permitted, and variable modifications of methionine oxidation and N-terminal acetylation were allowed. “Match between runs” was used to maximise peptide detection. The false discovery rate was set at 0.01, using a target-decoy based search applied at both peptide and protein group levels (38). Proteins identified as contaminants and false positives were removed. Protein abundance values for each sample were generated using the label-free quantification (LFQ) method (39). Our mass spectrometry proteomics data have been deposited to the ProteomeXchange Consortium via the PRIDE (40) partner repository with the dataset identifier PXD032158. The Andromeda platform within MaxQuant generated protein groups and majority protein groups (MPGs). MPGs contain proteins sharing at least 50% of their peptides (38), increasing identification confidence. As such, only MPGs were considered in subsequent statistical analyses. Hereafter, where possible we use the term protein in exchange of MPG, as it provides a more intuitive biological description.

### Statistical Analyses and Functional Groupings of Plasma Proteins

To establish the extent of variation in protein abundances between the control and HSA-immunized sharks across the time course, principal component analyses (PCA) were performed in R using the prcomp function, and visualized using ggplot2 v3.2.1 (41). As there was only a single control shark, statistical analyses at the level of individual proteins were restricted to using the four immunized sharks, assuming protein levels at day 0 as an internal baseline control for each animal (14). We filtered the data to retain proteins with LFQ values present in at least 8 out of 12 timepoints in every immunized shark across the timecourse (n=260 proteins), ensuring the analyses were based on consistently identified peptides. Prior to applying this filter, 46.62% of 37,560 LFQ values represented zeros. After filtering, 15,600 LFQ values were retained, with just 1.21% (189 LFQ values) representing zeros. The 15,600 LFQ values were log2 transformed and the 189 missing values imputed using missForest, a random forest-based method (42). All values were normalized to day 0 to minimize the effects of differences in starting abundances prior to immunization.

Using the above dataset, we aimed to identify proteins showing the most repeatable changes in abundance in the different immunized shark individuals across the sampling timecourse. One-way ANOVA offers a simple method to identify the proportion of variability in protein abundance explained by sampling day (i.e., the ANOVA R^2^ statistic), such that proteins showing the highest R^2^ values are those where the highest proportion of protein abundance between individuals is explained by differences in sampling days (14). We thus performed one-way ANOVA in Minitab v20.3.0 individually for all 260 proteins, based on imputed values normalized to day 0, and using time as a fixed factor. As a cut-off, we retained proteins as candidates of interest where at least half of the variance (R^2^ >0.5) was explained by sampling day. We recorded if sampling day was significant at *P* ≤ 0.05, before and after Benjamini-Hochberg (BH) correction (260 separate analyses). Tukey’s test was used to assess differences in abundance across days for two proteins showing *P* ≤ 0.05 after BH correction. The Anderson-Darling test was performed to verify that the residuals of each model were normally distributed, and Levene’s test was used to assess homogeneity of variance.

Proteins showing the top 5% ANOVA R^2^ values were subjected to hierarchical clustering analysis, to determine and visualize at which stage of the time course their abundance changes occurred. This was performed in PermutMatrix (43) using mean values for the normalized log2 transformed and imputed abundance data. Clustering and seriation were based on Pearson’s correlation coefficient dissimilarity after z-score normalization. The multiple-fragment heuristic seriation method was used with complete linkage (furthest neighbour) aggregation to obtain hierarchical clusters.

STRING (44) was used to obtain an overview of functional groupings for human homologues to the nurse shark proteins. The sequences of the first proteins in each MPG in the filtered dataset were uploaded to https://string-db.org/, and used as BLASTp queries to obtain a list of the closest human homologues. Gene Ontology (GO) (45, 46) and Reactome (i.e., groups of molecules participating in biological pathways) (47) enrichment analyses were then performed for the human putative homologues within STRING, contrasting expectations against a background of all human proteins.

### Definition of Orthology Among Nurse Shark and Whale Shark Proteins

Orthofinder, a phylogenetic orthology inference software (48), was used to identify putative whale shark orthologs in the raw nurse shark plasma proteomic dataset. Predicted proteins from the *de novo* nurse shark transcriptome and the whale shark reference genome (ASM164234v2) were uploaded to OrthoFinder. To ensure that any nurse shark proteins lacking orthologs in both nurse and whale shark were not excluded from the OrthoFinder output, protein sequences from additional vertebrate species were also uploaded, These included predicted proteins from the sea lamprey (*Petromyzon marinus*) genome (https://genomes.stowers.org/sealamprey: PMZ_v3.1 Proteins) and the hagfish (*Eptatretus burgeri*) genome (GCA_900186335.2), and all NCBI RefSeq proteins for the elephant shark (*Callorhinchus milii*), spotted gar (*Lepisosteus oculatus*), northern pike (*Esox lucius*), rainbow trout, zebrafish (*Danio rerio*), and human (*Homo sapiens*). Species vs. species all-protein BLASTp and reciprocal BLASTp hits were normalized for sequence length, avoiding bias towards poor quality hits for longer sequences over good quality hits for short sequences, and towards sequences in more closely related species over sequences in more distantly related species (i.e., normalizing for phylogenetic distance). The resulting reciprocal best normalized hits (RBNH) were used to determine inclusion within an orthogroup. For inclusion, each of a pair of genes in any two species must either be a RBNH or have a BLASTp bit-score greater than the lowest RBNH for either gene. The results were then analyzed based on proteins identified proteomically using the nurse shark transcriptome as the protein reference database, and considering only orthologs identified in nurse shark and whale shark.

### Nurse Shark Plasma Proteins Annotated as ‘Uncharacterized’

Fifty of the proteins in the filtered dataset were annotated as ‘uncharacterized’. To better understand their putative roles, each was used in manual BLASTp searches against the NCBI RefSeq protein database for Chondrichthyes, to identify homologues previously annotated in other shark species (cut-off values: coverage ≥65%; ID ≥50%; e-value <0.0001). Where Chondrichthyes homologues were also annotated as ‘uncharacterized’, additional BLASTp searches, using the same cut-off criteria, were performed against the NCBI RefSeq protein database for all taxa excluding cartilaginous fishes, to identify homologues in other evolutionary lineages.

### Domain Predictions, Sequence Alignment, and Phylogenetic Analysis

Conserved domains were identified by submission of protein sequences to the NCBI Conserved Domain database. Protein sequences were tested for the presence of signal peptides by submission to https://services.healthtech.dtu.dk/service.php?SignalP, and transmembrane domains by submission to https://services.healthtech.dtu.dk/service.php?TMHMM-2.0 and https://phobius.sbc.su.se/cgi-bin/predict.pl. Phylogenetic analysis was used to clarify the evolutionary relationships of two nurse shark proteins, annotated as uncharacterized in the *de novo* transcriptome. These proteins were used as queries for BLASTp searches against RefSeq proteins for all taxa in NCBI, with hits filtered to proteins showing >65/50% sequence coverage/identity to the query (e-value <1e^-04^). They were also submitted to the NCBI conserved domain database, which revealed that both proteins possessed the same secreted novel AID/APOBEC-like deaminase 1 (SNAD1) superfamily domain. Attempting to establish their identity, the nurse shark proteins were added to a representative sample of proteins used in a previous analysis of AID/APOBEC-like deaminases performed by Krishnan etal. (49). Sequence alignment was performed using MAFFT v.7 (50). Trees were built using the IQ-TREE maximum likelihood method (51, 52), which estimated and employed the best fitting amino acid substitution model (53). Ultra-fast bootstrapping (54) was used to generate branch support values. Consensus trees were visualised and rendered in Mega X (55).

## Results

### Generation of a High-Quality Nurse Shark Transcriptome Assembly

A multi-tissue transcriptome for nurse shark, inclusive of multiple immune organs, was generated using PacBio and Illumina technologies (details in **Supplementary Tables S1, S2**, respectively). We generated ~710 million paired-end clean Illumina reads (**Supplementary Table S1**), along with 33,737 high quality PacBio transcripts following clustering and polishing (**Supplementary Table S2**). These sequences were combined and used for *de novo* transcriptome assembly using a hybrid short- and long-read assembly algorithm (**Supplementary Table S3**). Following clustering and ORF prediction, the final transcriptome comprised 62,682 sequences that contained an ORF >80 amino acids, of which 45,314 were annotated with a UniProt protein (**Supplementary Table S3**). Transcriptome completeness was assessed using BUSCO v.3.0.2 (34, 35) against the vertebrata Odb10 database. 83.5% complete orthologs were recovered (**Supplementary Table S4**), comparing favourably to the most recent whale shark genome assembly (56), which showed a completeness of 78.7% using the same approach. This confirmed that our new transcriptome is an appropriate reference database for high-throughput proteomics in nurse shark, while further representing a useful resource available for future investigations.

### Nurse Shark Immunizations and Plasma LC-MS/MS

To gain a comprehensive overview of the plasma proteome, we sampled blood plasma from five sharks, in both immunized and unimmunized states. Four nurse sharks (hereafter Purple, Red, Yellow, and Green) were immunized with HSA/CFA, with an additional shark being sham-immunized with PBS. Non-lethal blood sampling of all sharks was performed prior to immunization (day 0) and on days 1, 2, 3, 5, 7, 14, 21, 28, 35, 42, and 49 post-immunization.

**Figure 1:**
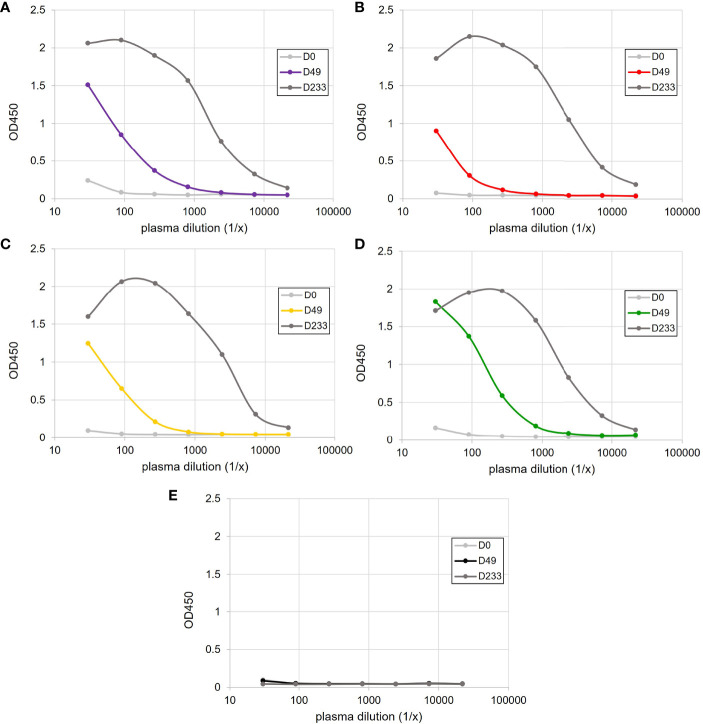
Antigen-specific IgM binding ELISAs following the immunization of four sharks, **(A)** Purple, **(B)** Red, **(C)** Yellow and **(D)** Green, with HSA-CFA and **(E)** a single sham-immunized shark. Plasma samples were taken prior to immunization (D0) and on days 49 (D49) and 233 (D233) post-immunization.

Antigen-binding ELISAs were performed on day 0 and day 49 plasma to measure increases in HSA-specific Ig. While different response magnitudes were observed across individuals, only the HSA-immunized sharks showed an increase in target-specific IgM between day 0 and day 49 (**Figures 1A–E**). After additional boosting following the end of this study, and then plasma sampling on day 233, antigen-specific IgM titres had peaked in all HSA-immunized animals (**Figures 1A–D**), indicating that each animal had indeed established a robust adaptive response.

All 60 plasma samples were subjected to LC-MS/MS analysis, using the predicted proteins from our nurse shark transcriptome as the reference. We identified 626 multiple protein groups (MPGs) comprising 1,754 individual proteins. Individual proteins assigned to an MPG typically represent isoforms, or potentially the products of recently duplicated genes (14).

### Overview of Nurse Shark Plasma Protein Functions

STRING (57) identified 409 human homologues to the 626 proteins identified against the nurse shark transcriptome, of which 299 had unique annotations (**Supplementary Table S5**) and contributed to 297 biological processes (**Supplementary Table S6**). The lower number of human homologues identified, compared to the number of nurse shark proteins, is likely due to some of the nurse shark proteins being either splice or transcript variants. MaxQuant assigns proteins to an MPG according to their peptide content, with proteins sharing ≥50% of their peptides being grouped in the same MPG. Minor differences in amino acid content can therefore lead to proteins being grouped in different MPGs even if they are the same proteins. For instance, contigs Seq28890 and Seq39578 were both identified in our transcriptome as being most similar to the whale shark protein annotated as XP_020390559 (Apo B-100) but are located in different MPGs. In addition, some nurse shark proteins could be sufficiently similar to the genes underpinning the human protein annotations to have met the BLASTp criteria to be considered as a human homologue but have <50% shared peptide content and therefore be assigned to separate MPGs. In our previous paper (14), we identified an ancestral Apo A-1/Apo A-IV-like protein which diverged into Apo A-I and Apo A-IV after the divergence of bony fishes. Ancestral proteins such as this may also be sufficiently similar to their diverged descendants to meet the BLASTp criteria. This situation likely applies to additional proteins, but would require clarification *via* comprehensive phylogenetic analysis.

Of the 299 human homologues with unique annotations, 62 were associated with coagulation, including coagulation factors, prothrombin, fibrinogen, plasminogen, and heparin. A further 59 were associated with complement, including C1 complex molecules (C1q, C1r, and C1s), C2/factor B, C3, C4, C5, C6, C7, C8, C9, the positive complement regulator properdin, and inhibitory factors H and I. Homologues of SERPINs and other protease inhibitors were also identified, such as alpha-1-antitrypsin, alpha-2-antiplasmin, plasma protease C1 inhibitor, and protein Z-dependent protease inhibitor. Homologues of human enzymatic proteins identified in nurse shark plasma included sulfhydryl oxidase, prolyl endopeptidase, aminopeptidase, and glutathione peroxidase (**Supplementary Table S5**).

### Uncharacterised Nurse Shark Plasma Proteins

Fifty nurse shark proteins (out of 626) were annotated as ‘uncharacterized’. BLASTp searches against the NCBI RefSeq protein database for Chondrichthyes revealed that 35 proteins shared homology with small-spotted catshark (*Scyliorhinus canicula*) or white shark (*Carcharodon carcharias*) proteins annotated as Igs (**Supplementary Table S7**). Two further proteins showed homology to proteins annotated as dynein axonemal-associated protein 1 (in whale shark) and neural cell adhesion molecule L1 (in white shark) (**Supplementary Table S7**). Three proteins did not satisfy the cut-off, but their top BLASTp hits in Chondrichthyes were annotated variously as zinc finger protein 239-like, tumor necrosis factor receptor superfamily member 3-like, and immunoglobulin mu heavy chain-like (**Supplementary Table S7**).

The ten remaining proteins were annotated as ‘uncharacterized’ in all searched Chondrichthyes species. BLASTp queries against the non-redundant NCBI RefSeq protein database identified homologues in other evolutionary lineages for four of the ten proteins. Two proteins had homologues in invertebrates and fishes (encoded by contigs Seq1466 and 44204), and two in fishes and amphibians (encoded by contigs Seq9619 and 18791), which were all annotated as ‘uncharacterized’. Notably, none of the ten nurse shark putative proteins shared significant homology with any mammalian protein (**Supplementary Table S8**).

**Figure 2:**
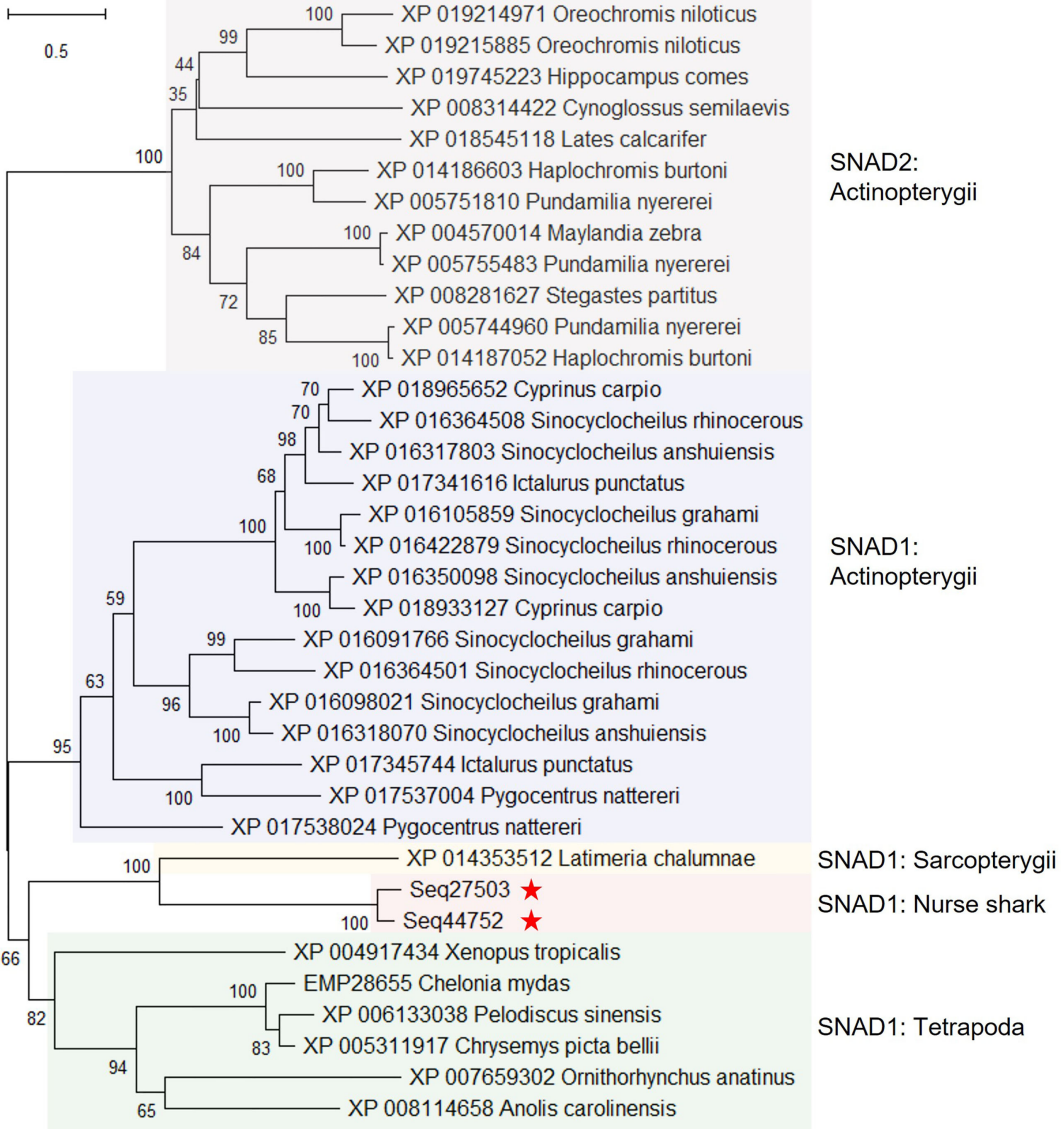
Maximum likelihood consensus tree of selected SNAD1 and SNAD2 sequences from 49, and predicted proteins from nurse shark contigs Seq27503 and Seq44752. The tree, which is rooted to the SNAD2 clade, was generated using the best fitting amino acid substitution model (WAG+F+I+G4) and includes branch support values from 1,000 ultrafast bootstrap replicates.

Conserved domain searches revealed domains in 4 of the 10 uncharacterised proteins (**Supplementary Table S9**). Two proteins (encoded by contigs Seq27503 and 44752) possessed the same SNAD1 superfamily domain (**Supplementary Table S9**) thus far only found in a small family of potentially secreted AID/APOBEC-like deaminases. Given this, we added these nurse shark proteins to a representative sample of proteins used in a previous analysis of AID/APOBEC-like deaminases (49), attempting to establish their identity. Phylogenetic analysis revealed that the proteins encoded by Seq27503 and Seq44752 identified in nurse shark plasma are indeed SNAD1 orthologs (**Figure 2**). Among the other uncharacterized proteins, one (encoded by contig Seq34341) contains domains for peptidase C80 family, Ca2+-binding protein, and RTX toxin-related (COG2931), while another (encoded by contig Seq38071) harbours copper/zinc superoxide dismutase (SOD and SODC) domains (**Supplementary Table S9**).

### Changes in the Nurse Shark Proteome Following Immune Challenge

**Figure 3:**
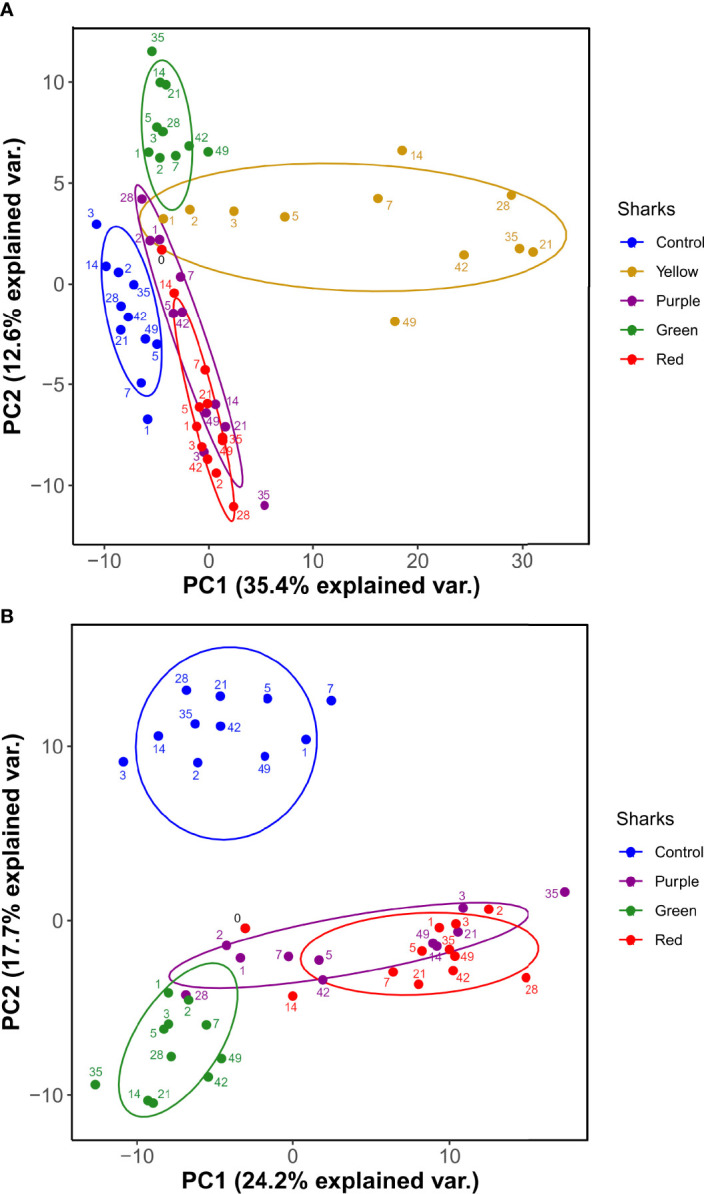
PCA of nurse shark plasma proteome (260 protein dataset) **(A)** for all sharks and **(B)** following the exclusion of shark ‘Yellow’. Ellipses are 95% confidence intervals around the centroid.

PCA was used to visualize variation between the control and HSA-immunized sharks considering the 260 proteins across the sampling time course. One immunized shark (Yellow) contributed a large proportion of the variation along PC1 (**Figure 3A**), suggesting a major difference in plasma protein abundance changes compared to the other sharks. During routine checks it was noted that Yellow had developed a granuloma-like lump at the immunization site, which was not observed in the other sharks. A second PCA with Yellow removed (i.e., using 48, rather than 60, plasma samples) showed that the variation along PC2 (17.7%) was largely composed of the differences between the sham-immunized control and remaining HSA-immunized sharks (**Figure 3B**). However, there were also individual-specific differences between the remaining HSA-immunized sharks, primarily along PC1, explaining an even larger proportion of variation (**Figure 3B**). Subsequent statistical analyses excluded Yellow due to our concerns regarding the distinct response compared to the remaining three immunized sharks.

### Proteins Showing the Most Repeatable Response to Immunization

**Figure 4:**
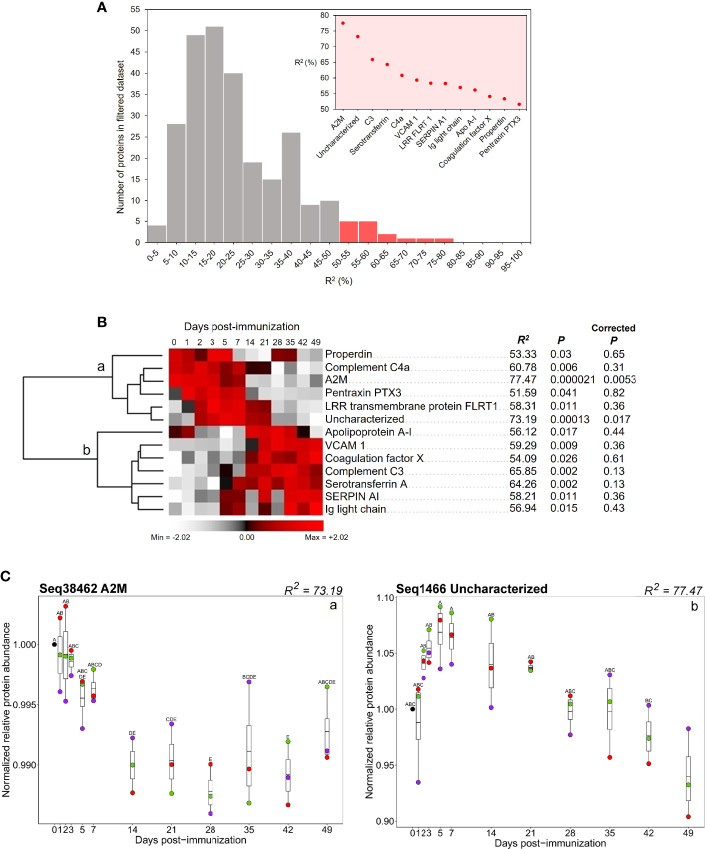
**(A)** Distribution of ANOVA R^2^ values for all proteins in the 260 protein dataset. Red bars indicate the distribution of proteins with the top 5% of R^2^ values. Inset shows R^2^ values for proteins within the top 5% of R^2^ values. **(B)** Hierarchical clustering of 13 proteins comprising the top 5% of R^2^ values. Values for R^2^, *P*, and corrected *P*, for each protein, are provided. **(C)** Abundance profiles for two proteins within the set comprising the top 5% of R^2^ values (i.e., **Figure 3A**) that showed *P* ≤ 0.05 after BH correction (a) protein translated from transcriptome contig Seq38462 (annotated as A2M); (b) protein translated from Seq1466 (annotated as uncharacterized). Contig numbers for protein annotations: properdin: Seq19272; complement C4a: Seq39499; A2M: Seq38462; pentraxin PTX3: Seq24909; LRR transmembrane protein: Seq2356; uncharacterized: Seq1466; apolipoprotein A-I: Seq1759; VCAM 1: Seq31639; coagulation factor X: Seq7348; complement C3: Seq36113; serotransferrin A: Seq3561; SERPIN AI: Seq7563; Ig light chain: Seq12648. Different letters shown on the plots indicate days with significantly different protein abundance values (Tukey’s test).

To identify proteins showing similar changes in abundance across the post-immunization time course in three sharks (Purple, Red, and Green), we ranked the 260 proteins remaining after quality filtering (see Methods) by the proportion of variance explained by sampling day, represented by the ANOVA R^2^ statistic (**Figure 4A**; **Supplementary Table S10**) (after 14). We took 13 proteins comprising the top 5% of R^2^ values as those showing the most repeatable response to immunization in our dataset (**Figure 4A**). For these proteins, R^2^ ranged from 51.6-77.5% (i.e., more than half of the variance in protein abundance across individuals was explained by sampling day). After BH correction for multiple testing, just two of these proteins showed *P <*0.05 (**Figure 4B**).

Hierarchical clustering of the 13 prioritized proteins divided them into two groups, showing highest respective abundances early (i.e., days 0-7) or later in the time course (between days 14-49) (**Figure 4B**, group a and b, respectively). Group ‘a’ included the two proteins that showed the highest R^2^ values (A2M and protein predicted from contig Seq1466). Levels of A2M, an inhibitory factor for the complement and coagulatory systems (58, 59) were highest in all immunized sharks between day 0 and day 7, before decreasing in abundance between day 7 and day 28 (**Figure 4B**, group ‘a’; **Figure 4C**, ‘a’). The protein predicted from contig ‘Seq1466’ was annotated “uncharacterized”. Abundance of this 146aa protein increased in all immunized sharks from day 0 to 5, before decreasing between days 5 and 49 (**Figure 4B**, group a; **Figure 4C**, ‘b’). This protein was used as a query for BLASTp searches against RefSeq proteins for all taxa in NCBI. This revealed homologous proteins in invertebrate, cephalochordate, chondrichthyan and teleost species (≥70% coverage, ≥50% identity, and e-value <0.0001), all of which were also annotated as ‘uncharacterized’ (**Supplementary Tables S7, S8**). No conserved domains were found in contig Seq1466 nor its putative homologues, so the nature and functional role of this protein remains unclear.

Four additional proteins annotated as properdin, complement C4a, pentraxin PTX3 and LRR transmembrane protein FLRT1 showed highest abundances during the first week post-immunization (**Figure 4B**, group ‘a’). Mean levels of properdin, a positive regulator for the alternative complement pathway (60) were highest between days 0-5, decreased to day 21, increased during days 28-35 and finally decreased to the end of the time course. Anaphylatoxin C4a, produced following cleavage of C4 in the complement classical and lectin pathways (61), had highest abundance between days 0-7, after which it decreased. The pattern recognition molecule pentraxin 3 (PTX3) (62) increased between day 0 and day 1, remaining at higher abundances until day 7, then decreased. The leucine-rich repeat (LRR) transmembrane protein FLRT1, which functions as an extracellular matrix protein in mammals (63), increased at day 2, remained at higher levels until day 21, after which it decreased to the end of the time course.

In group ‘b’, seven proteins showed highest abundances between days 14-49 (**Figure 4B**, group ‘b’). Levels of a protein annotated as Apo A-IV, associated in mammals with lipid transport, but which may play an immunological role in fishes (14), increased at day 1, then decreased to day 7. This was followed by a second increase at day 14 and further decreases on days 42 and 49. Vascular cell adhesion molecule-1 (also known as VCAM1 or CD106), the soluble form of which is associated with chronic inflammatory diseases such as rheumatoid arthritis in mammals (64, 65), coagulation factor X, and complement C3 abundances all increased in the immunized sharks at day 14 and remained at elevated levels until the end of the time course. Serotransferrin A, an iron transport protein (66), started to increase on day 5 and attained its highest levels between days 7 and 49 (**Figure 4B**, group ‘b’). Levels of the serine protease inhibitor SERPIN AI (67) increased on day 1, reaching its highest levels between days 5 and 49, albeit with decreases on days 14 and 28 (**Figure 4B**, group ‘b’). Ig light chain levels also increased at day 5, remaining at elevated levels until the end of the time course, although with 2 separate decreases in abundance on days 14 and 28 (**Figure 4B**, group ‘b’).

### Characterizing the Nurse Shark Plasma Proteome Using Predicted Proteins in the Whale Shark Genome

While the number of high-quality genomes is rapidly increasing there are still many species across phylogeny yet to be sequenced, including most elasmobranchs. Therefore, as well as generating a species-specific annotation of proteins for our proteomic analysis of nurse shark, we decided to compare our results to a different strategy assuming the absence of a reference transcriptome. To this end, we mapped our LC-MS/MS data against the predicted proteins from the whale shark genome (37). Nurse shark and whale shark are members of the same Order, the Orectolobiformes (carpet sharks), but are evolutionarily separated by approximately 100 million years (68). Here, 297 MPGs containing 477 proteins were identified, 47.4% and 27.2% less, respectively, than when using the nurse shark transcriptome.

**Figure 5:**
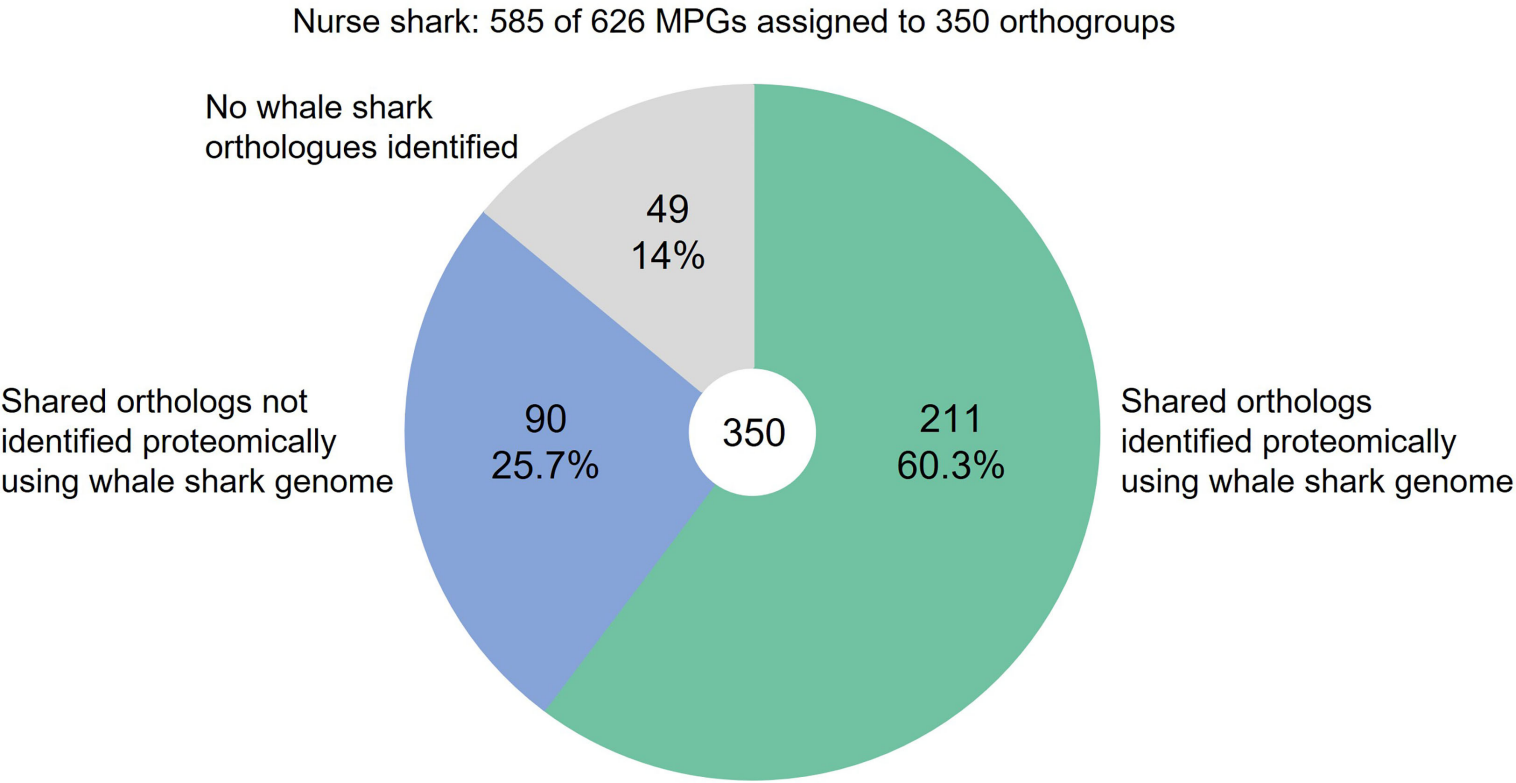
Proportion of nurse shark plasma proteins detected when using the whale shark genome as the reference database compared with the species-matched transcriptome.

OrthoFinder was then used to identify orthologous proteins in the nurse shark and whale shark (**Supplementary Table S11**), so that the extent of protein identification using the two reference databases could be compared. Of the 626 proteins in the nurse shark raw proteomic dataset, 585 were assigned to 350 orthogroups (i.e., groups containing proteins with orthologs in either the nurse shark alone, or in both species) (**Figure 5**). Of the 350 nurse shark orthogroups, 301 had orthologs in the whale shark, among which 211 were identified during proteomic analysis using the whale shark genome as the reference protein database (**Figure 5**). These 211 orthologs included immune proteins such as apolipoproteins A-IV and B-100, complement components C1-C9, factor I, and properdin, and Igs. Of the 139 nurse shark orthogroups which were not identified during proteomic analysis using the whale shark protein database, 90 did have orthologs in the whale shark (**Figure 5**). Those 90 proteins included hemopexin, pentraxin PTX3, and plasma protease C1 inhibitor. Forty-nine nurse shark proteins had no whale shark orthologs, including A2M, complement factor H, and hepcidin.

## Discussion

The study of immune function in diverse species, sampled from across phylogeny, is key to understanding the evolution of immune protection and the exploration of lineage- and species-specific traits. Ambitious efforts such as the Earth Biogenome project (69) are rapidly increasing the number of draft genomes; however, the provision of species-specific reagents for immunology studies lags significantly. For example, while the nurse shark is arguably the most developed model for the study of immunity in cartilaginous fishes, functional studies remain severely hampered by a paucity of specific mAbs. To overcome these limitations, in this study we applied MS-based proteomics to nurse shark plasma to identify the proteins present and quantify abundance changes following immunization. Most proteomic studies rely upon an annotated, species-matched genome as the reference database for protein identification (e.g., 13, 14, 27). As the nurse shark genome has yet to be sequenced, we compared two alternate approaches for protein identification. First, we generated a high quality, multi-tissue nurse shark transcriptome to use as our reference database. This strategy permitted the identification of 626 nurse shark plasma proteins, vastly improving our knowledge of the plasma proteome in cartilaginous fishes. Among the proteins identified were the small handful of previously characterized nurse shark plasma proteins, including the heme-clearing protein hemopexin (26) as well as key mediators of humoral immunity such as Igs (4) and almost the entire repertoire of shark complement system proteins (70). Now supplementing this are an extensive repertoire of immune, coagulation, and metabolic plasma proteins, previously unexamined in cartilaginous fishes. For example, our discovery of SNAD1 proteins in nurse shark plasma proves these molecules have a longer evolutionary history than previously thought (49), having evolved prior to the emergence of jawed vertebrates rather than in a bony vertebrate ancestor. Most AID/APOBEC-like family members function by converting cytidine to uridine in single-stranded nucleic acids and so generally exert their function within cells. Further, the high evolutionary rate observed for SNAD1 sequences, especially at loops implicated in substrate binding, indicates pathogen-driven selection. Together, this suggests that SNADs are delivered from the plasma into virally infected cells, perhaps *via* endocytosis, where they exert antiviral activity by mutating viral genomes, e.g., as has previously been observed for mammalian APOBEC3s acting upon HIV (71). However, it is the group of previously ‘uncharacterized’ plasma proteins that pique our interest the most. Approximately half of the (non-Ig) uncharacterized nurse shark proteins have homologues in other species, all of which are also uncharacterized at this time, while the remainder appear to be more restricted in their distribution (potentially species- or lineage-specific molecules). It is likely that many more evolutionarily ancient proteins, which have been lost from mammals (e.g., SNAD1), but which remain in cartilaginous fishes and other non-mammalian species, have been overlooked. The combination of LC-MS/MS proteomics and species-specific protein reference databases generated from high quality genomic/transcriptomic data permit the detection of such proteins.

In our second approach, we used the annotated draft genome of a related shark species, the whale shark, as our reference database. Despite approximately 100 million years of evolutionary separation between the two shark species (68), this strategy still permitted the identification of 297 nurse shark plasma proteins, i.e., roughly half the number identified with our nurse shark transcriptome. Among the proteins identified were several apolipoproteins and coagulation factors, in addition to key immune mediators including complement cascade components and Igs. Undoubtedly the evolutionary separation between the chosen species will affect the amount of data obtained and, given that protein identification is based upon peptide content, this strategy will be biased towards the identification of slowly evolving proteins that remain highly conserved between species. This was reinforced by our data, where 90 out of 350 proteins orthologous to both nurse shark and whale shark were not detected proteomically using the whale shark genome as a reference database. It is likely that divergence of primary protein sequences prevented proteomic identification in these cases. However, while a species-matched reference database is certainly preferable, our data show it is possible to use a reference database from a related species, should the sampled species lack a draft genome or comprehensive transcriptome.

While there was considerable variation in response between the immunized sharks – unsurprising given these are unrelated, outbred animals - several proteins showed highly repeatable responses (i.e., more than half of the variance in protein abundance between individuals was explained by sampling day) across the post-immunization time course in all animals. These included several complement system proteins (namely C3, anaphylatoxin C4a, properdin, and pentraxin 3), the coagulation factor X, and the broad-range protease inhibitor A2M that can inhibit both the coagulation and complement cascades (58, 59). Nurse shark Apo A-IV was also among this group, aligning with our previous proteomic study of rainbow trout plasma that showed several apolipoproteins exhibited highly consistent changes in abundance following immunization (14). The Apo A-IV abundance profiles are also very similar between rainbow trout and nurse shark, strongly supporting a yet unidentified role for apolipoproteins in immune protection in these lineages.

Of special interest among the proteins with the most repeatable responses is an as yet completely uncharacterized plasma protein (Seq1466). With a predicted molecular weight of ~15kDa and no recognizable domains, homologues of this nurse shark protein were also found in invertebrates, cephalochordates, and teleost fishes. Together our data suggest a protein with ancient origins and an important immune function, presumably lost from tetrapods, ripe for further exploration.

As a final thought, this study clearly shows the power of high-resolution proteomics as a discovery tool and transformative technology for the study of immune responses in diverse species. It has also highlighted many new targets for future exploration in nurse sharks (and other species). However, there remains room for improvement in such approaches; a primary limitation of data-dependent proteomics is a bias towards the detection of the most abundant molecules (72). Thus, many key immune proteins that are usually present at lower abundances (e.g., cytokines) were not detected in this study. Alternative methods of proteomic analysis, such as sequential window acquisition of all theoretical fragment-ion spectra (SWATH) (73, 74), detect proteins across a much wider dynamic range than the method used here, and are less biased towards the detection of abundant proteins (74). Lower abundance proteins may therefore be more readily identified and quantified, providing important additional information regarding the immune response.

## Conclusions

Our *de novo* transcriptome has greatly increased the genetic resources for nurse shark, providing a comprehensive and well-annotated set of transcripts and proteins to underpin future work in this species. This is also the first time a high-resolution proteomic approach has been used to catalogue the shark plasma proteome and evaluate how it changes following immune challenge. Our data provide a robust dataset of reliably identified immune-relevant proteins in chondrichthyans, which can be explored in future studies.

## Data Availability Statement

The datasets presented in this study can be found in online repositories. The names of the repository/repositories and accession number(s) can be found below: ProteomeXchange, PXD032158. NCBI, PRJNA841433.

## Ethics Statement

The animal study was reviewed and approved by The University of Maryland, School of Medicine Institutional Animal Care and Use Committee (IACUC).

## Author Contributions

FKB, DJM, and HD designed the study; HD and HM conducted animal work, performed binding ELISAs, and prepared samples for sequencing/proteomics; FKB and MKG assembled and annotated the nurse shark transcriptome; FKB and DAS performed proteomics lab work; FKB performed proteomic data analysis and prepared figures/tables; FKB, DJM, and HD performed data interpretation and drafted the manuscript. All authors contributed to the article and approved the final manuscript.

## Funding

FKB was supported by a BBSRC-funded EASTBIO Doctoral Training Partnership studentship (grant number BB/M010996/1) awarded to HD. HM is supported by NIH-NIAID predoctoral fellowship F31AI147532. DM received institutional strategic funding from the BBSRC (grants: BBS/E/D/10002071 and BBS/E/D/20002174). For the purpose of open access, the author has applied a Creative Commons Attribution (CC BY) licence to any Author Accepted Manuscript version arising from this submission.

## Conflict of Interest

The authors declare that the research was conducted in the absence of any commercial or financial relationships that could be construed as a potential conflict of interest.

## Publisher’s Note

All claims expressed in this article are solely those of the authors and do not necessarily represent those of their affiliated organizations, or those of the publisher, the editors and the reviewers. Any product that may be evaluated in this article, or claim that may be made by its manufacturer, is not guaranteed or endorsed by the publisher.
